# Racial disparities in prevalence, screening, and complications of osteoporosis in patients with inflammatory bowel disease: a retrospective cohort study

**DOI:** 10.1093/crocol/otag032

**Published:** 2026-04-26

**Authors:** Benjamin Eric Hoffman, Jennifer Youn, Madeline Alizadeh, Tamiru Berake, Ki Jung Lee, Lisa Ceglia, Sonia Friedman

**Affiliations:** Department of Medicine, Tufts Medical Center, Boston, MA, United States; Department of Medicine, Tufts Medical Center, Boston, MA, United States; Institute for Genome Sciences, University of Maryland Baltimore, Baltimore, MD, United States; Department of Medicine, Tufts Medical Center, Boston, MA, United States; Department of Medicine, Tufts Medical Center, Boston, MA, United States; Division of Endocrinology, Diabetes, and Metabolism, Tufts Medical Center, Boston, MA, United States; Division of Gastroenterology, Tufts University School of Medicine, Boston, MA, United States

**Keywords:** inflammatory bowel disease, osteoporosis, osteopenia

## Abstract

**Introduction:**

Osteoporosis is a complication of inflammatory bowel disease (IBD) with an estimated prevalence of 18%-42%. Given the paucity of data, this exploratory study aims to identify racial differences in prevalence, screening, and complications of osteoporosis in patients with IBD.

**Methods:**

A retrospective cohort study was conducted for patients with IBD and treated at Tufts Medical Center from January 1, 2010 to December 31, 2023. The rates of osteoporosis, osteopenia, and osteoporotic fractures were calculated and compared between White, Black, and Asian patients. Dual energy X-ray absorptiometry (DXA) screening rates were compared between the three races. Fisher’s exact test was used to compare categorical variables, and Welch’s t-test was used to compare quantitative data. Multivariable regression analysis was performed to assess the relationship between risk factors and the presence of osteoporosis, osteoporotic fractures, and cumulative events. Glucocorticoid exposure was defined as having had one or more glucocorticoid prescriptions during the study period.

**Results:**

A total of 2517 patients with IBD were identified. There were 175 (7.0%) Asian, 156 (6.2%) Black, and 2186 (86.8%) White patients. There were statistically higher rates of osteoporosis and osteoporotic fractures in Asian patients compared to Black and White patients, respectively; (17.1% vs. 10.9% vs. 9.8%, *P* = .013) and (10.9% vs. 5.1% vs. 6.0%, *P* = 0.049). Black patients had higher rates of glucocorticoid exposure compared to Asians and Whites (39.1% vs. 28.6% vs. 27.2%, *P* = 0.007). There were no statistically significant differences in DXA screening rates between races.

**Discussion:**

Asian populations with IBD were found to have higher prevalence of osteoporosis and osteoporotic fractures; these differences may reflect unmeasured genetic, environmental, or socioeconomic factors.

## Graphical Abstract

**Graphical Abstract:**
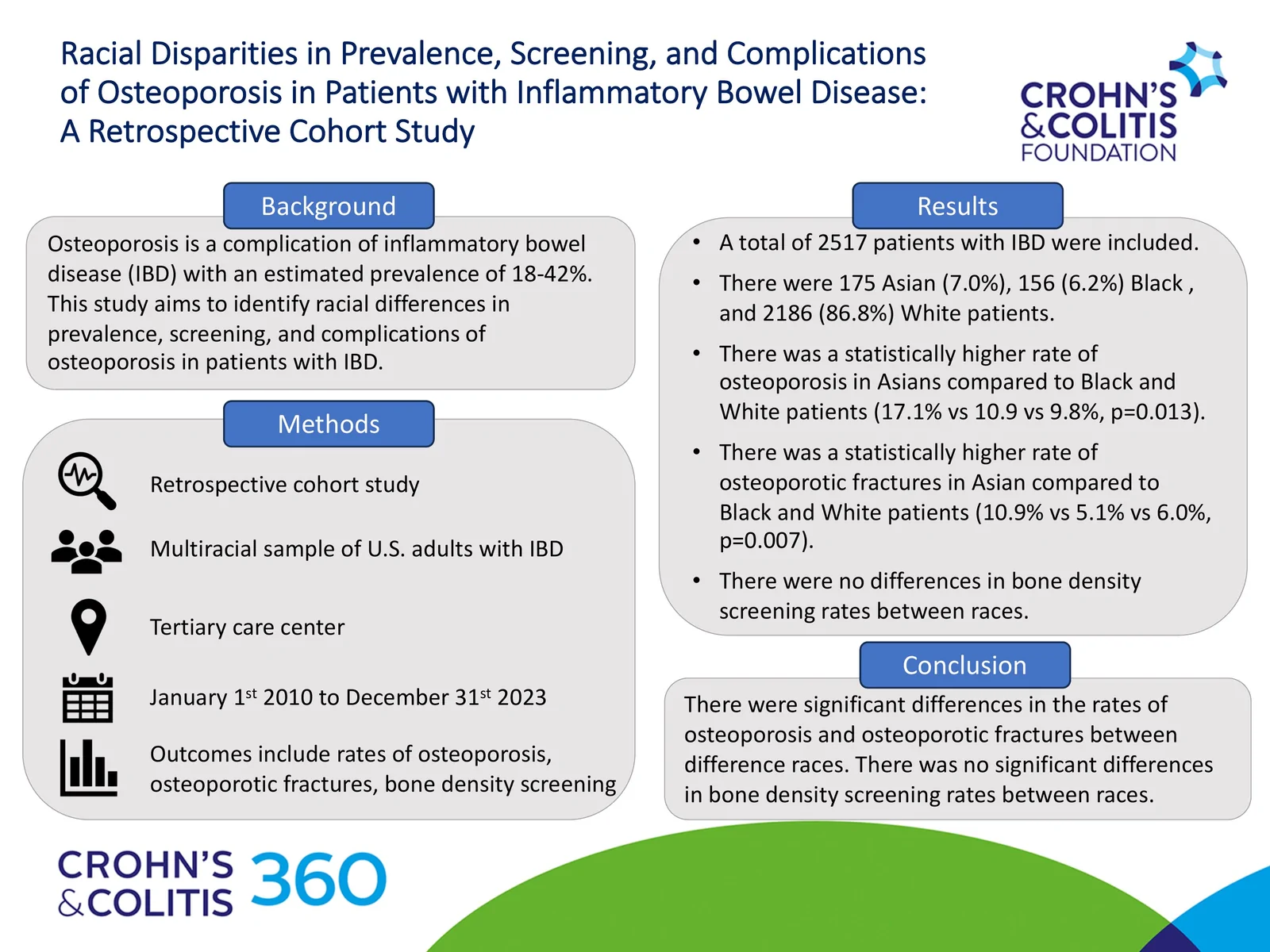

## Introduction

Inflammatory bowel disease (IBD) is a chronic idiopathic inflammatory disease of the gastrointestinal tract and includes Crohn’s disease (CD) and ulcerative colitis (UC). The incidence and prevalence of IBD is increasing globally, particularly in recently industrialized countries.^1^^,^^2^ For example, East Asia has experienced a marked increase in the incidence of IBD over time. In Hong Kong, the incidence of UC and CD has increased by 16-fold and 146-fold, respectively, since 1981 over the span of 33 years.^3^ In South Korea, the incidence of UC and CD has increased 20-fold and 40-fold, respectively, over a 30-year period.^4^ South-East Asia and the Middle East have also seen recent increases in both the incidence and prevalence of IBD, an observation made in parallel with the regions’ trends toward industrialization.^5^ The prevalence of IBD among Asian Americans is more recently estimated to be 403 per 100 000 persons.^6^ In addition to geographic variation, racial and ethnic differences in IBD presentation and complications have been described.^7–9^ However, studies investigating bone health disparities across racial groups in U.S. IBD cohorts are lacking.^10^

Osteoporosis, a metabolic bone disorder characterized by low bone mass and skeletal fragility, is a common comorbid condition seen in patients with IBD.^10^ The prevalence of osteoporosis in IBD patients is estimated to be 18%-42%.^11^ The development of osteoporosis in IBD patients is thought to be multifactorial, including altered bone metabolism in the setting of systemic inflammation, exposure to glucocorticoids, and nutritional deficiencies in vitamin D and calcium.^12–14^ Screening for osteoporosis for those at increased risk is performed by use of dual energy X-ray absorptiometry (DXA), which measures bone mineral density. In some cases, patients are first diagnosed with osteoporosis with initial encounters of fragility fractures, which are fractures that occur in the setting of low or minimal trauma and are diagnostic of osteoporosis. These fractures are associated with significant morbidity, mortality, and increased health care costs.^15–17^ Studies have shown that the incidence of osteoporotic fractures is increased in IBD patients compared to the general population.^18^^,^^19^

We use the term race/ethnicity recognizing it is a social construct that reflects complex interactions of genetics, environment, and socioeconomic factors. Previous studies have described racial differences in the prevalence and screening of osteoporosis in the general U.S. population, including lower screening rates among Black women and lower bone mineral density in Asians compared to other ethnic groups.^20^ These findings raise the question of whether these differences extend to patients with IBD. Our study aimed to better characterize racial differences in the prevalence, screening, complications, and associated risk factors of osteoporosis among IBD patients in a multiracial sample of U.S. adults.

## Methods

An exploratory retrospective cohort study was conducted for all adult patients with a diagnosis of UC or CD and treated in either inpatient or outpatient settings at Tufts Medical Center (TMC) in Boston, Massachusetts, from January 1, 2010 to December 31, 2023. For all patients, follow up was included up to their last recorded encounter within the healthcare system. Patients that were lost to follow up were not excluded from the study. Patients with a diagnosis of UC or CD were extracted from the TMC data warehouse and stored in the Observational Medical Outcomes Partnership (OMOP) common data model format. The OMOP data model uses a standardized data structure to organize healthcare datasets in standardized vocabularies such as ICD codes, Systematized Nomenclature of Medicine (SNOMED) codes, and RxNorm codes.^21^ All clinical events of interests, including the demographics and diagnoses, were collected using either ICD-10 codes or SNOMED vocabulary (Table S2). Medication prescriptions were collected using RxNorm codes (Table S3).

All patients with an established diagnosis of UC or CD and self-identifying as White, Black, or Asian were included for analysis. Patient demographic data were obtained, including sex, current age at the time of analysis, and race. Patients were categorized based on self-identified race, either by birth or descent. Asian patients included patients identifying as Asian, Asian Indian, or South Asian. Social history data, including smoking history and alcohol history were obtained. Smoking status documented closest to the IBD diagnosis date was obtained and defined by four categories of never, former, current, and unknown. Alcohol history documented closest to the IBD diagnosis date was obtained and defined by four categories of never, former, current, and unknown. IBD duration in years was defined as time between IBD diagnosis and time of analysis.

Additional baseline clinical characteristics were obtained including menopausal status and corticosteroid exposure status. Those who were postmenopausal were identified by documented diagnosis of postmenopause or history of oophorectomy. Corticosteroid exposure status was defined as having had at least one prescription of oral or IV corticosteroid (Table S2) during the study period. Cumulative corticosteroid dose over the study period for each participant was calculated by summation of the prednisone-equivalent dosing of all corticosteroid prescriptions for each individual, with 10.0 mg of prednisone set as equivalent to 8.0 mg of methylprednisolone, 40.0 mg of hydrocortisone, 3.0 mg of budesonide, and 0.02 mg of fludrocortisone.^22^^,^^23^ Median cumulative corticosteroid dosing with interquartile range (IQR) was calculated for each race. Vitamin D levels, measured as 25-OH vitamin D, were collected at measurement closest to date of IBD diagnosis. DXA screening was defined as the presence of any documented DXA scan during the study period.

The prevalence of osteoporosis was calculated as the percentage of patients with documented diagnosis of osteoporosis. The prevalence of osteopenia was calculated as the percentage of patients with documented diagnosis of osteopenia. The prevalence of osteoporotic fractures included patients with all documented fractures at sites associated with low-energy trauma (excluding foot, skull, and facial fractures). Categories of osteoporotic fractures include vertebral columns, rib, humerus, forearm, femur/hip, wrist, and hand fractures.^24^ Metabolic bone disease event (MBDE) was defined as having the occurrence of at least one of the following diagnoses: osteoporosis, osteopenia, or osteoporotic fracture. The time in years from IBD diagnosis date to first instance of MBDE for each individual was calculated. Lastly, the DXA screening rates were calculated as a percentage of patients with documented DXA scan results during the study period.

Patient demographic, social, and clinical characteristics were compared across racial groups, Welch’s t-test and Fisher’s Exact test used for comparing continuous (ie median, IQR) and categorical variables (ie ratios), respectively. Values of *P* < .05 were considered statistically significant. Post hoc Fisher’s multiple comparisons were performed for sub-group comparisons when applicable. Multivariable logistic regression models were developed using Generalized Linear Modeling (GLM) with binomial distribution to compare those with vs. without osteoporosis, osteoporotic fractures, and all osteoporosis associated variables (MBDE), and constructed to include variables of interest and potential confounders. A significance threshold of α = 0.05 was utilized, and 95% confidence intervals (CI) were calculated. All data processing and statistical analyses were conducted using the “stats,” “RVAideMemoire,” and “vctrs” packages in R software version 4.1.3 (R Foundation for Statistical Computing, Vienna, Austria).

## Results

### Study population and characteristics

A total of 2517 patients with IBD were treated at TMC in both inpatient and outpatient settings between January 1, 2010 to December 31, 2023. Patients were followed until the end of the study period or death, whichever came first. Among the cohort, 2186 (86.8%) were White, 156 (6.2%) were Black, and 175 (6.9%) were Asian (Table 1). There were 87 (49.7%) Asian females, 86 (55.1%) Black females, and 1141 (52.2%). White females were included in the cohort, and there was not a statistically significant difference in sex among the three racial groups. Mean age at time of analysis was similar between racial groups, with mean age being in the latter half of the fourth decade of life (Asians 49.9 years, Blacks 47.3 years, and Whites 49.5 years, *P* = .359). Regarding IBD type, CD comprised 48.0% of Asians, 53.2% of Blacks, and 53.5% of Whites (*P* = .378). The difference in mean IBD disease duration was statistically significant between the three racial groups, with 6.3 years for Asians, 7.5 years for Blacks, and 7.4 years for Whites (*P* = .006). Black participants trended toward higher rates of current smoking status compared to Asians and Whites, respectively (13.4% vs. 8.3% vs. 8.0%; *P* = .057). Asians were statistically significantly less likely to report current alcohol use compared to Black and White participants (21.7% vs. 36.5% vs. 45.2%; *P* < .001). Serum 25-OH vitamin D levels were not significantly different and were low across participants of all races (Asians 13.1 ng/mL, Blacks 18.1 ng/mL, and Whites 18.8 ng/mL, *P* = .115). Black participants had a significantly higher percentage of glucocorticoid exposure during the study period compared to that of Whites and Asians (39.1% vs. 27.1% vs. 28.5%, respectively, *P* = .007). Median cumulative steroid dosing between races during the study period was not significantly different (Asians 400 mg, Blacks 300 mg, Whites 323 mg, *P* = .928) (Table 1). Among females, postmenopausal status was not significantly different between races (Asians 10.3%, Blacks 7.1%, and Whites 7.9%, *P* = .461).

**Table 1:** Demographics and characteristics of study population by race.

	**Asian (*n* = 175)**	**Black (*n* = 156)**	**White (*n* = 2186)**	***P*-value**
**Sex**				.617
** Female**	87 (49.71%)	86 (55.13%)	1141 (52.19%)	
**Male**	88 (50.29%)	70 (44.87%)	1045 (47.81%)	
**Mean age (median [IQR1, IQR4])**	49.9 (46.0, [31.5, 69.0])	47.3 (46.5, [32.0, 62.0])	49.5 (47.0, [34.0, 64.0])	.359
**IBD type**				.378
**CD**	84 (48.00%)	83 (53.21%)	1169 (53.47%)	
**UC**	91 (52.00%)	73 (46.79%)	1016 (46.48%)	
**Mean IBD disease duration in years (median [IQR1, IQR3)**	6.3 (6.0, [3.0, 10.0])	7.5 (8.0, [4.0, 11.0])	7.4 (8.0, [4.0, 11.0])	.006
**Smoking**				.057
**Never**	122 (69.72%)	95 (60.90%)	1315 (60.16%)	
**Former**	30(17.14%)	29 (18.59%)	502 (22.96%)	
**Current**	14(8.00%)	21 (13.46%)	182 (8.33%)	
**Unknown**	9(5.14%)	11 (7.05%)	187 (8.55%)	
**Alcohol**				≤.001
**Never**	75(42.86%)	51 (32.69%)	501 (22.92%)	
**Former**	51(29.14%)	34 (21.80%)	464 (21.23%)	
**Current**	38(21.71%)	57 (36.54%)	988 (45.20%)	
**Unknown**	11(6.29%)	14 (8.97%)	233 (10.66%)	
**Serum**	*n* = 54	*n* = 49	*n* = 524	
**Vitamin D2+D3 in ng/mL**	13.1 (9.0, [2.0, 18.0])	18.1 (9.0, [2.0, 25.0])	18.8 (14.0, [2.0, 31.0])	.115
**Postmenopausal^a^**	18 (10.29%)	11(7.05%)	172(7.87%)	.461
**Steroid exposure**	50(28.57%)	61(39.10%)	594(27.17%)	.007
**Median cumulative prednisone equivalent dose for those who had steroids in milligrams [IQR], [range]**	400 [105, 971], [10, 3567]	300 [90, 738], [10, 5629]	323 [80, 865], [3, 7876]	.928

a Includes those with a documented diagnosis of post-menopause or history of oophorectomy.

### Prevalence of osteoporosis, osteopenia, and related comorbidities by race

Prevalence of osteoporosis, osteopenia, and related comorbidities were compared across Asian, Black, and White participants. Asian individuals demonstrated the highest prevalence of osteoporosis (17.1%) compared to Black (9.8%) and White (10.9%) individuals (*P* = .013; Table 2) and higher rates of osteoporotic fractures (10.9% vs. 5.1% vs. 6.0%, respectively, *P* = .049). While not statistically significant, Black individuals had higher prevalence of osteopenia (4.5%) compared to Asian (1.1%) and White (2.0%) individuals (*P* = .107; Table 2). Asians were more likely to have MBDE compared to Black and White patients (23.4% vs. 17.9% vs. 14.2%, respectively, *P* = .007). With respect to IBD type, osteoporosis was observed in 16.7% of Asians with CD, 17.6% of Asians with UC, 9.6% of Blacks with CD, 12.3% of Blacks with UC, 10.0% of Whites with CD, and 9.5% of Whites with UC (*P* = .110; Table S1). The majority of individuals in each racial group had IBD diagnoses before the first occurrence of MBDE (Asians 80.5%, Blacks 75%, and Whites 68.2%, *P* = .157). The mean time in years from IBD diagnosis to MBDE did not differ significantly between race (Asians 3.76 years, Blacks 4.22 years, and Whites 3.91 years, *P* = .441). For the minority of individuals with MBDE occurrence before diagnosis of IBD, the mean time in years to IBD diagnosis was less than 3 years for each race and was not statistically significant. There was not a statistically significant difference in DXA screening rates between races (Table 2).

**Table 2:** Osteoporosis and related comorbidities among different races in patients with IBD.

	Asian (*n* = 175)	Black (*n* = 156)	White (*n* = 2186)	*P*-value
**Osteoporosis**	30 (17.14%)	17 (10.90%)	214 (9.79%)	.013
**Osteopenia**	2 (1.14%)	7 (4.49%)	44 (2.01%)	.107
**Osteoporotic fractures**	19 (10.86%)	8 (5.13%)	132 (6.04%)	.049
**MBDE**	41 (23.43%)	28 (17.95%)	311 (14.23%)	.007
**DEXA screening**	56 (32.00%)	52 (33.33%)	693 (31.70%)	.901
	*n* = 41	*n* = 28	*n* = 311	
**IBD diagnosed prior to MBDE**	33 (80.5%)	21 (75.00%)	212 (68.17%)	.157
**Time to MBDE diagnosis following IBD diagnosis (median, [IQR], [range])**	3.76 [1.33, 5.48] [0.25, 13.29]	4.22 [2.81, 8.52] [1.28, 12.26]	3.91 [1.95, 6.73] [0.01, 13.46]	.441
**Time to IBD diagnosis in years for those with MBDE diagnosed prior to IBD (median, [IQR], [range])**	2.88 [1.46, 3.65] [0.80, 6.45]	2.24 [1.12, 5.71] [0.00, 8.03]	1.43 [0.43, 2.78] [0.00, 8.60]	.102

### Characteristics and risk factors of patients with osteoporosis

The study cohort was subsequently analyzed based on the presence (n = 261, 10.4%) or absence (n = 2256, 89.6%) of osteoporosis (Table 3). Significantly more participants with osteoporosis compared to those without osteoporosis were female (73.5%, *P* < .001), older aged (68.3 years vs. 47.2 years, *P* < 0.001), postmenopausal (23.7% vs. 6.1%, *P* < 0.001) and were more likely to be either current or former smokers at the time of IBD diagnosis (4.9%, 40.2% vs. 9.0%, 20.2%, respectively; *P* < .001). Mean vitamin D levels in the osteoporosis group were statistically lower than those in the non-osteoporosis group (14.8 ng/mL vs. 19.0 ng/mL, *P* < .001). Glucocorticoid exposure was significantly higher in the osteoporosis group compared to the non-osteoporosis group (44.4% vs. 26.1%, respectively, *P* < .001). There was a statistically significant difference in mean cumulative steroid dosing between the osteoporosis and non-osteoporosis groups (902 mg vs. 658 mg, respectively, *P* < .001) while median cumulative dosing was similar between the 2 groups (318 mg vs. 325 mg, respectively). Similarly, significantly more participants with osteoporotic fractures compared to those without fractures were female (60.3% vs. 51.6%, *P* = .033), postmenopausal (23.7% vs. 6.96%, *P* < .001), current or former smokers at the time of IBD diagnosis (7.5%, 42.1% vs. 8.6%, 20.9%, respectively, *P* < 0.001), and had lower mean vitamin D levels (12.8 ng/mL vs. 18.9 ng/mL, *P* < .001) (Table 4). Patients with positive osteoporotic fracture history were also more likely to have reported current or former alcohol use compared to those without history of osteoporotic fracture (74.2% vs. 64.2%, respectively, *P* < 0.001). Presence of glucocorticoid exposure was more likely to be associated with osteoporotic fractures compared to those without fractures (42.1% vs. 27.0% respectively, *P* < .001). Mean cumulative steroid dose was not significantly different between fracture and non-fracture subgroups (904 mg vs. 677 mg, respectively, *P* = .174).

**Table 3:** Demographics and characteristics of study population by presence of osteoporosis.

	**Osteoporosis (*n* = 261)**	**No osteoporosis (*n* = 2256)**	***P*-value**
**Sex**			≤.001
**Female**	192 (73.56%)	1122 (49.73%)	
**Male**	69 (26.44%)	1134 (50.27%)	
**Mean age (Median [IQR])**	68.3 (71.0 [60.0, 79.0])	47.2 (44.0 [32.0, 61.0])	≤.001
**IBD type**			≥.999
**CD**	139 (53.26%)	1197 (53.06%)	
**UC**	122 (46.74%)	1058 (46.90%)	
**Mean IBD disease duration (median [IQR])**	8.6 (9.0 [5.0, 13.0])	7.2 (7.0 [3.0, 11.0])	≤.001
**Smoking**			≤.001
**Never**	123 (47.13%)	1409 (62.46%)	
**Former**	105 (40.23%)	456 (20.21%)	
**Current**	13 (4.98%)	204 (9.04%)	
**Unknown**	20 (7.66%)	187 (8.29%)	
**Alcohol**			.002
**Never**	74 (28.35%)	553 (24.51%)	
**Former**	76 (29.12%)	473 (20.97%)	
**Current**	87 (33.33%)	996 (44.15%)	
**Unknown**	24 (9.20%)	234 (10.37%)	
**Serum**	*n* = 115	*n* = 512	≤.001
**Vitamin D2+D3 in ng/mL**	14.8 (2.0 [2.0, 24.5])	19.0 (15.0 [2.0, 31.0])	
**Postmenopausal^a^**	62 (23.75%)	139 (6.16%)	≤.001
**Steroid exposure**	116 (44.44%)	589 (26.11%)	≤.001
**Median cumulative prednisone equivalent dose for those who had steroids in milligrams [IQR], [range]**	318 [50, 1492], [3, 6626] Mean: 902	325 [90, 800], [3, 7876] Mean: 658	≤.001

a Includes those with a documented diagnosis of post-menopause or history of oophorectomy.

**Table 4:** Demographics and characteristics of study population by presence of osteoporotic fracture.

	**Osteoporotic fractures (*n* = 159)**	**No osteoporotic fractures (*n* = 2358)**	***P*-value**
**Sex**			.033
**Female**	96 (60.38%)	1218 (51.65%)	
**Male**	63 (39.62%)	1140 (48.35%)	
**Mean age (median [IQR])**	64.8 (67.0 [55.5, 76.0])	48.4 (45.0 [33.0, 63.0])	≤.001
**IBD type**			.743
**CD**	82 (51.57%)	1254 (53.18%)	
**UC**	77 (48.43%)	1103 (46.78%)	
**Mean IBD disease duration years (median [IQR])**	7.7 (8.0 [4.0, 11.0])	7.3 (7.0 [3.0, 11.0])	.252
**Smoking**			≤.001
**Never**	71 (44.65%)	1461 (61.96%)	
**Former**	67 (42.14%)	494 (20.95%)	
**Current**	12 (7.55%)	205 (8.69%)	
**Unknown**	9 (5.66%)	198 (8.40%)	
**Alcohol**			≤.001
**Never**	31 (19.50%)	596 (25.28%)	
**Former**	57 (35.85%)	492 (20.86%)	
**Current**	61 (38.36%)	1022 (43.34%)	
**Unknown**	10 (6.29%)	248 (10.52%)	
**Serum**	*n* = 68	*n* = 559	≤.001
**Vitamin D2+D3 in ng/mL**	12.8 (2.0 [2.0, 20.8])	18.9 (14.0 [2.0, 31.0])	
**Postmenopausal^a^**	37 (23.27%)	164 (6.96%)	<.001
**Steroid exposure**	67 (42.14%)	638 (27.06%)	≤.001
**Median cumulative prednisone equivalent dose for those who had steroids in milligrams [IQR], [range]**	373 [77 1152], [35 628] Mean: 904	325 [80 858], [37 876] Mean: 677	.174

a Includes those with a documented diagnosis of post-menopause or history of oophorectomy.

### Multivariable regression analysis

Considering the potential for impact of several factors concomitantly on osteoporosis development, we performed multivariate logistic regression to assess the association between osteoporosis, related factors, and variables of interest (eg race, sex, smoking status). In comparing those with versus without osteoporosis, female sex (OR = 2.7, *P*-value < 0.001), former smoking status (OR = 2.3, *P*-value = .015), older age (OR = 1.1, *P*-value < .001), and Asian race relative to White race (2.0, *P*-value = .011) remained statistically significantly associated with osteoporosis while alcohol use and menopausal status no longer were (Table 5). Similarly, when comparing those with versus without a history of osteoporotic fractures (Table 6), older age (OR = 1.04, *P*-value < .001), and Asian race relative to White race (OR = 2.1, *P*-value = .007) remained statistically significantly associated, as did Asian race relative to Black race (OR = 2.6, *P*-value = .047), and postmenopausal status (OR = 1.8, *P*-value = .018). When comparing MBDE (Table 7), female sex (OR = 1.8, *P*-value = .002), former smoking status (OR = 1.7, *P*-value = .038), older age (OR = 1.1, *P*-value < .001), Asian race relative to White race (OR = 1.9, *P*-value = .005), postmenopausal status (OR = 1.5, *P*-value = .027), and former alcohol use (OR = 1.4, *P*-value = .039) remained significantly associated with outcomes in the multivariate model.

**Table 5:** Assessment of the relationship between osteoporosis and associated factors using logistic multivariate regression.

Factor	**OR**	**CI (95%)**	***P*-value**
**Male (relative to F)**	0.37	[0.26, 0.53]	≤.001
**Former smoker (relative to current)**	2.26	[1.21, 4.59]	.014
**Never smoker (relative to current)**	1.77	[0.96, 3.55]	.087
**Former alcohol use (relative to current)**	1.22	[0.85, 1.77]	.275
**Never alcohol use (relative to current)**	1.12	[0.76, 1.64]	.562
**Age**	1.07	[1.06, 1.08]	≤.001
**Black race (relative to Asian)**	0.73	[0.34, 1.54]	.411
**White race (relative to Asian)**	0.51	[0.30, 0.87]	.011
**Postmenopausal (compared to pre)**	1.30	[0.86, 1.94]	.204
**Cumulative steroid dose**	1.00	[1.000, 1.001]	≤.001

**Table 6:** Assessment of the relationship between osteoporotic fracture and associated factors using logistic multivariate regression.

Factor	**OR**	**CI (95%)**	***P*-value**
**Male (relative to F)**	0.85	[0.57, 1.26]	.408
**Former smoker (relative to current)**	1.52	[0.80, 3.16]	.225
**Never smoker (relative to current)**	1.10	[0.58, 2.26]	.790
**Former alcohol use (relative to current)**	1.32	[0.88, 1.97]	.179
**Never alcohol use (relative to current)**	0.69	[0.42, 1.10]	.126
**Age**	1.04	[1.03, 1.05]	≤.001
**Black race (relative to Asian)**	0.38	[0.14, 0.95]	.047
**White race (relative to Asian)**	0.47	[0.27, 0.83]	.007
**Postmenopausal (compared to pre)**	1.79	[1.10, 2.90]	.018
**Cumulative steroid dose**	1.00	[1.000, 1.001]	≤.001

**Table 7:** Assessment of the relationship between MBDE and associated factors using logistic multivariate regression.

Factor	**OR**	**CI (95%)**	***P*-value**
**Male (relative to F)**	0.55	[0.41, 0.73]	.002
**Former smoker (relative to current)**	1.71	[1.05, 2.90]	.038
**Never smoker (relative to current)**	1.47	[0.91, 2.45]	.129
**Former alcohol use (relative to current)**	1.38	[1.02, 1.86]	.039
**Never alcohol use (relative to current)**	0.91	[0.65, 1.27]	.590
**Age**	1.06	[1.05, 1.07]	≤.001
**Black race (relative to Asian)**	0.88	[0.46, 1.66]	.697
**White race (relative to Asian)**	0.52	[0.35, 0.83]	.005
**Postmenopausal (compared to pre)**	1.51	[1.05, 2.18]	.027
**Cumulative steroid dose**	1.00	[1.000, 1.001]	≤.001

## Discussion

Our study found that Asians with IBD have a statistically higher prevalence of osteoporosis compared to Black and White participants with IBD. Interestingly, Asians were found to have less glucocorticoid exposure compared to Blacks despite this observation as well as no significant differences in age, sex, smoking rates, vitamin D levels, and post-menopausal status when compared to Blacks and Whites. Asian patients were also found to have higher rates of osteoporotic fractures compared to Black and White patients (*P* = .049). The prevalence of osteoporosis in the study cohort was observed to be near 10%, which is slightly higher than rates observed in patients with IBD irrespective of race (4%-9%) in a recent systematic review by Kärsand et al.^25^ Both advanced age and menopausal status, well-known risk factors for osteoporosis,^26^^,^^27^ were found to increase the risk of osteoporosis among all three racial groups. Interestingly, the rates of osteoporosis and fractures were highest in Asian individuals despite lower active and former alcohol use, which is another known risk factor for osteoporosis.^27^ While not statistically significant, Asians had lower rates of smoking, which is another known risk factor for osteoporosis.^27^ These observations may suggest Asian race as an independent risk factor for osteoporosis in IBD patients. Possible explanations for these observations include genetic disposition, differences in dietary habits, including calcium and vitamin D intake, lower baseline bone mineral density, or unmeasured socioeconomic factors.

Given our long study period and inclusion of individuals at different times according to first documentation of IBD diagnosis, there were significant variabilities in follow up times for each individual. Reassuringly, the time to diagnosis of MBDE from date of IBD diagnosis did not differ significantly between racial groups (Table 2). Similarly, for the minority of individuals in each racial group with recorded instance of MBDE prior to diagnosis of IBD, the time to IBD occurrence was relatively short and occurred within 3 years. It is possible that these individuals with occurrence of MBDE prior to IBD diagnosis were screened according to general population screening recommendations and may have already had underlying IBD that was yet to be formally diagnosed.

U.S.-based studies examining racial disparities in the prevalence and complications of osteoporosis among patients with IBD are limited. Previous studies investigating the association between IBD and osteoporosis in Asian populations were based in East Asia with varying results. A Korean-based study by Yang et al. observed a non-statistically significant higher prevalence of osteoporosis in patients with IBD compared to the general population.^28^ Kang et al. examined genetic influences on osteoporosis development in European and East Asian populations with IBD and did not find a genetic correlation or causation between IBD and osteoporosis in the East Asian population.^29^ Genetic influences were also investigated by Xu et al. and, in contrast, found CD to have a causal relationship with osteoporosis in East Asian populations.^30^ A Taiwanese study by Tsai et al. found a positive association between IBD and osteoporosis.^31^ Our study is the first U.S.-based cohort to investigate the racial differences in the prevalence of osteoporosis and its related complications in patients with IBD.

Osteoporotic fractures are the dreaded complication of osteoporosis, and patients with IBD are at increased risk.^32^ Our study found Asians to have higher rates of osteoporotic fracture, which is in contrast to findings in a recent review by Lo et al. that showed lower rates of hip fractures in Asian populations compared to non-Hispanic White populations.^33^ DXA screenings for osteoporosis remain important in the primary prevention of osteoporotic fractures, and the current screening guidelines for patients with IBD are the same as those for the general population.^34^ Given the observed higher rates of both osteoporosis and fractures in the Asian population in our study, an earlier screening strategy for this population should be considered. Our study did not find a significant difference in screening rates between races, however, disparities in screening, particularly among Black patients, have been observed in non-IBD cohorts in a previous study.^35^

Notably, Black patients in our study were observed to have higher rates of glucocorticoid exposure compared to White and Asian patients (39.1% vs. 27.1% vs. 28.5%, respectively, *P* = .007). Despite the observed racial differences in glucocorticoid exposure, defined as having had at least one glucocorticoid prescription during the study period, the median total prednisone-equivalent cumulative dose was not significantly different between the three racial groups. This implies Blacks are more likely to be prescribed steroids, but at similar cumulative dosing as that of Whites and Asians.

Mean vitamin D levels were low among all races and ranged from 13.1-18.8 ng/mL which is notably lower than the consensus recommended range of 20-50 ng/mL for optimal skeletal health.^36^ These differences in mean vitamin D levels between races were not statistically significant. In addition to traditional risk factors for vitamin D deficiency, including poor sunlight exposure and nutritional deficiency, CD-related risk factors, including small bowel resection and inflammation, are thought to contribute to vitamin D deficiency in these patients.^37^ Although a recent systematic review found higher prevalence of vitamin D deficiency in CD patients, and vitamin D deficiency has been linked to osteoporosis,^25^ our study showed that there was no statistically significant difference in the proportion of participants with CD in the osteoporosis and non-osteoporosis groups.

Our study had several strengths. As mentioned above, this is the first U.S.-based study investigating the prevalence of osteoporosis and osteoporotic fractures in patients with IBD. We were able to include a large and diverse cohort of 2517 participants representing the real-world patient population in an urban city. We were able to obtain data over a 13-year period from 2010 to 2023. Additionally, the database used in this study was stored in a standardized OMOP common data model and uses standardized vocabulary such as ICD-10 codes, SNOMED codes, and RxNorm codes, which allowed consistent and accurate data extraction across a long period of time. All patients with at least one encounter within our institution’s healthcare system were included, limiting potential attrition bias in our study. Lastly, we were able to extract many different variables, including standard demographic data and potential cofounding variables: steroid exposure, menopausal status, vitamin D levels, smoking status, and alcohol use status.

We recognize several limitations in our study. As this is a single-center study, the degree of generalizability is less than that of multi-center studies. Body mass index (BMI) was a notable confounding variable that was unable to be extracted and included in our analysis. This was primarily due to limitations in height documentation in patients’ electronic medical records. BMI is known to be associated with increased risk for both osteoporosis and osteoporotic fractures.^38^^,^^39^ Additionally, it is known that Asian Americans tend to have lower BMI compared to Whites^40^ and may have contributed to the observation of Asian individuals having higher prevalence of osteoporosis and osteoporotic fractures in this study. IBD phenotypes and activity were not collected as due to the size of the cohort, we did not perform individual chart review. Given the retrospective nature of the study, we were unable to determine immigration status of participants and acknowledge this distinction may help to evaluate genetic vs. environmental influences on our observed disparities. We lacked data on socioeconomic status, including income, insurance coverage, and education status, which may confound associations attributed to race/ethnicity. Vitamin D levels were collected at the time closest to diagnosis of IBD and may not reflect baseline levels over time. Additionally, data on vitamin D supplementation, receptor activator of nuclear factor kappa-B ligand inhibitors, and biologics were incomplete; these therapies may influence outcomes. The mechanism of trauma for fractures were not recorded.

As our cohort was identified through the use of ICD-10 codes, there remains the possibility that not all eligible patients were included in the study if a particular diagnosis code was inappropriately omitted from their medical chart. Given the nature of a retrospective study, some data points may be missing or unavailable. Additionally, we recognize that individuals who were lost to follow-up may have experienced outcomes that were not accounted for, leading to potential underestimation of prevalence rates. Regarding fractures, imaging was not reviewed in this study, and it is possible that incidental fractures identified in these reports were not then appropriately coded as an osteoporotic fracture in patient charts.

Cumulative steroid dose in prednisone equivalent was used in this study to quantify attributable steroid risk to osteoporosis development. Traditionally, this has been examined in the context of duration of therapy, with greater than 3 months indicating significant risk.^41^ We were unable to quantify the duration of steroid therapy for patients given the end dates for steroid prescriptions were unavailable in our institution’s database. Therefore, the cumulative glucocorticoid dose was used instead as a risk factor for osteoporosis.

Future studies are needed to further investigate the relationship between Asian race and osteoporosis in those with IBD. Given multiple comparisons, findings should be interpreted as exploratory and hypothesis-generating. In addition to building upon existing genetic investigations into this association,^29^^,^^30^ cultural influences, such as dietary habits should be investigated among Asian Americans, as East Asians are known to have low dietary calcium intake.^42^

## Conclusion

To the best of our knowledge, this is the first U.S.-based study investigating racial differences in osteoporosis among IBD populations. Our study demonstrated increased prevalence of osteoporosis and osteoporotic fractures in an Asian-American population with IBD, an overall understudied population with regard to the intersection of IBD and osteoporosis. Screening differences between races were not present in our study. Vitamin D levels were low in the entire cohort. Further U.S.-based studies are needed to characterize the interaction between environment and race in IBD patients with regard to the development of osteoporosis in Western populations. Clinicians should be aware of the influence of race on rates of osteoporosis and osteoporotic fractures in the IBD population. Screening strategies for osteoporosis in Asian Americans with IBD may include recommending earlier DXA scans.

## Supplementary Material

otag032_Supplementary_Data

## Data Availability

Data are not publicly available.
