# Supplementary material for: Racial disparities in prevalence, screening, and complications of osteoporosis in patients with inflammatory bowel disease: a retrospective cohort study
Source: Crohns Colitis 360. 2026 Apr 26;8(2):otag032. doi: 10.1093/crocol/otag032 (PMC13161559; doi:10.1093/crocol/otag032)
Supplement: otag032_Supplementary_Data [file otag032_supplementary_data.docx]

Supplementary Table 1. Osteoporosis and related comorbidities among different races by IBD type

|  | Asians with CD (n=84) | Blacks with CD (n=83) | Whites with CD (n=1169) | Asians with UC  (n=91) | Blacks with UC  (n=73) | Whites with UC  (n=1016) | P value |
| --- | --- | --- | --- | --- | --- | --- | --- |
| Osteoporosis | 14 (16.7%) | 8 (9.6%) | 117 (10.0%) | 16 (17.6%) | 9 (12.3%) | 97 (9.5%) | 0.110 |
| Osteoporotic fractures | 8 (9.5%) | 6 (7.2%) | 68 (5.8%) | 11 (12.1%) | 2 (2.7%) | 64 (6.3%) | 0.172 |
| MBDE | 19 (22.6%) | 15 (18.1%) | 165 (14.1%) | 21 (23.1%) | 13 (17.8%) | 146 (14.4%) | 0.083 |

Supplementary Table 2. List of ICD-10 codes and SNOWMED codes

| **Diagnosis** | **SNOMED Code/ICD-10 Code** |
| --- | --- |
| Crohn’s Disease | 34000006/K.50 |
| Ulcerative Colitis | 64766004/K51 |
| Osteoporosis | 64859006 |
| Osteopenia | 312894000 |
| Osteoporotic fracture | 443165006 |
| **Wrist/Hand/Finger Fractures** |  |
| Closed Barton's fracture | 307713000 |
| Closed Colles' fracture | 269083002 |
| Closed fracture finger middle phalanx | 208450001 |
| Closed fracture hamate, hook | 208369005 |
| Closed fracture of base of fifth metacarpal | 698056009 |
| Closed fracture of base of metacarpal bone other than first metacarpal | 54641008 |
| Closed fracture of carpal bone | 9468002 |
| Closed fracture of distal phalanx of finger | 76865005 |
| Closed fracture of distal phalanx of index finger | 703986000 |
| Closed fracture of distal phalanx of ring finger | 704001001 |
| Closed fracture of fifth metacarpal | 704003003 |
| Closed fracture of hamate bone of wrist | 37174005 |
| Closed fracture of metacarpal bone | 208394006 |
| Closed fracture of middle phalanx of ring finger | 704163004 |
| Closed fracture of neck of fifth metacarpal bone | 704080002 |
| Closed fracture of phalanx of finger | 24424003 |
| Closed fracture of phalanx of middle finger | 704156004 |
| Closed fracture of phalanx of ring finger | 704155000 |
| Closed fracture of proximal phalanx of little finger | 704161002 |
| Closed fracture of proximal phalanx of middle finger | 704160001 |
| Closed fracture of scaphoid bone of wrist | 42818005 |
| Closed fracture of shaft of metacarpal bone | 46422008 |
| Closed fracture of triquetral bone of wrist | 30632004 |
| Closed fracture thumb distal phalanx | 208439004 |
| Closed fracture thumb proximal phalanx | 208434009 |
| Fracture at wrist and/or hand level | 208388003 |
| Fracture distal phalanx of thumb | 297132000 |
| Fracture malunion - hand | 287075005 |
| Fracture of carpal bone | 82065001 |
| Fracture of distal phalanx of finger | 36778005 |
| Fracture of hamate bone of wrist | 85922006 |
| Fracture of metacarpal bone | 208393000 |
| Fracture of middle phalanx of finger | 297131007 |
| Fracture of neck of fifth metacarpal | 263210007 |
| Fracture of phalanx of finger | 18171007 |
| Fracture of proximal phalanx of finger | 297130008 |
| Fracture of scaphoid bone of wrist | 31975004 |
| Fracture of triquetral bone of wrist | 7585008 |
| Open fracture of distal phalanx of index finger | 704187002 |
| Open fracture of distal phalanx of middle finger | 704188007 |
| Open fracture thumb distal phalanx | 208472004 |
| **Humerus/Forearm fractures** |  |
| Closed fracture distal humerus, lateral condyle | 208267005 |
| Closed fracture distal humerus, lateral epicondyle | 208271008 |
| Closed fracture of distal end of radius | 17222009 |
| Closed fracture of distal end of ulna | 50397009 |
| Closed fracture of forearm | 91419009 |
| Closed fracture of head of radius | 68854005 |
| Closed fracture of humerus | 43295006 |
| Closed fracture of lesser tuberosity of humerus | 703947004 |
| Closed fracture of lower end of humerus | 269080004 |
| Closed fracture of lower end of radius AND ulna | 33192001 |
| Closed fracture of neck of radius | 72497001 |
| Closed fracture of olecranon process of ulna | 64902007 |
| Closed fracture of proximal end of ulna | 33041006 |
| Closed fracture of radius | 111640008 |
| Closed fracture of shaft of humerus | 90235006 |
| Closed fracture of shaft of radius | 3228009 |
| Closed fracture of styloid process of ulna | 41036008 |
| Closed fracture of surgical neck of humerus | 73244003 |
| Closed fracture of upper end of humerus | 42636007 |
| Closed fracture olecranon, intra-articular | 208298007 |
| Closed fracture proximal humerus, greater tuberosity | 208242007 |
| Closed Monteggia's fracture | 29045004 |
| Closed torus fracture of radius | 429655000 |
| Closed transcondylar fracture of distal humerus | 704410001 |
| Fracture of distal end of humerus | 263192005 |
| Fracture of radius | 12676007 |
| Fracture of shaft of humerus | 50890004 |
| Fracture of surgical neck of humerus | 733405006 |
| Fracture of upper end of humerus | 127286005 |
| Fracture proximal phalanx of thumb | 297133005 |
| Open fracture of distal end of ulna | 649002 |
| Osteoporotic fracture of humerus | 704335006 |
| **Rib fractures** |  |
| Closed flail chest | 311408004 |
| Closed fracture of four ribs | 39335003 |
| Closed fracture of multiple ribs | 12204004 |
| Closed fracture of one rib | 45356009 |
| Closed fracture of rib | 60667009 |
| Closed fracture of seven ribs | 20121009 |
| Closed fracture of three ribs | 79546008 |
| Flail chest | 78011002 |
| Fracture of multiple ribs | 1261007 |
| Fracture of one rib | 20274005 |
| Fracture of rib | 33737001 |
| **Vertebral columns fractures** |  |
| Closed fracture lumbar vertebra | 207957008 |
| Closed fracture lumbar vertebra, burst | 207958003 |
| Closed fracture lumbar vertebra, wedge | 207959006 |
| Closed fracture of C1-C4 level with spinal cord injury | 8840000 |
| Closed fracture of cervical spine | 269062008 |
| Closed fracture of coccyx | 766775007 |
| Closed fracture of first cervical vertebra | 269063003 |
| Closed fracture of first lumbar vertebra | 11310841000119100 |
| Closed fracture of first thoracic vertebra | 11310521000119100 |
| Closed fracture of second cervical vertebra | 269064009 |
| Closed fracture of seventh cervical vertebra | 269069004 |
| Closed fracture of sixth cervical vertebra | 269068007 |
| Closed fracture of T7-T12 level with spinal cord injury | 72513001 |
| Closed fracture of third cervical vertebra | 269065005 |
| Closed fracture of vertebral column | 42157000 |
| Closed fracture sacrum | 207974008 |
| Closed fracture thoracic vertebra | 207938004 |
| Closed fracture thoracic vertebra, burst | 207939007 |
| Closed fracture thoracic vertebra, wedge | 207940009 |
| Compression fracture of lumbar spine | 426646004 |
| Compression fracture of vertebral column | 42942008 |
| Fracture of cervical spine | 125606003 |
| Fracture of fifth lumbar vertebra | 721498000 |
| Fracture of first cervical vertebra | 207983003 |
| Fracture of first lumbar vertebra | 721494003 |
| Fracture of first thoracic vertebra | 721415001 |
| Fracture of fourth lumbar vertebra | 721497005 |
| Fracture of lumbar spine | 125608002 |
| Fracture of odontoid process | 281910003 |
| Fracture of second cervical vertebra | 207984009 |
| Fracture of second lumbar vertebra | 721495002 |
| Fracture of seventh cervical vertebra | 721351009 |
| Fracture of sixth cervical vertebra | 721350005 |
| Fracture of third cervical vertebra | 721347007 |
| Fracture of third thoracic vertebra | 721417009 |
| Fracture of thoracic spine | 125607007 |
| Fracture of vertebral column | 50448004 |
| Open fracture lumbar vertebra, wedge | 207967003 |
| Osteoporotic fracture of vertebra | 11311601000119100 |
| Type II fracture of odontoid process of axis | 721345004 |
| Wedge fracture of lumbar vertebra | 281932007 |
| Wedge fracture of thoracic vertebra | 281922000 |
| **Femur/Hip** |  |
| Osteoporotic fracture of femur | 704330001 |

Supplementary Table 3. List of oral and IV corticosteroid prescriptions included in steroid use variable and calculations for cumulative steroid dosing

| Oral corticosteroid | RxNorm code | IV corticosteroid | RxNorm code |
| --- | --- | --- | --- |
| Prednisone | 8640 | Methylprednisolone | 6902 |
| Prednisolone | 8638 | Hydrocortisone | 5492 |
| Budesonide | 19831 | Dexamethasone | 3264 |
| Fludrocortisone | 4452 |  |  |
